# What's in a name? The case of cyanobacteria

**DOI:** 10.1111/jpy.12934

**Published:** 2019-11-15

**Authors:** Ferran Garcia‐Pichel, Jonathan P. Zehr, Debashish Bhattacharya, Himadri B. Pakrasi

**Affiliations:** ^1^ Center for Fundamental and Applied Microbiomics & School of Life Sciences Arizona State University Tempe Arizona 85287 USA; ^2^ Department of Ocean Sciences University of California Santa Cruz California 95064 USA; ^3^ Department of Biochemistry and Microbiology Rutgers University New Brunswick New Jersey 08901 USA; ^4^ Department of Biology Washington University St. Louis Missouri 63130 USA

**Keywords:** blue–green algae, Margulisbacteria, Melainabacteria, oxygenic photosynthesis, phylogenomics, Saganbacteria, Sericytochromatia

## Abstract

A redefinition of the cyanobacterial lineage has been proposed based on phylogenomic analysis of distantly related nonphototrophic lineages. We define Cyanobacteria here as “Organisms in the domain bacteria able to carry out oxygenic photosynthesis with water as an electron donor and to reduce carbon dioxide as a source of carbon, or those secondarily evolved from such organisms.”

The cyanobacteria, previously known as blue**–**green algae, have been, and still are, one of the most important microorganisms on Earth, and the subject of many biological, ecological, and evolutionary studies. They have also been the subject of lively nomenclatural discussions involving botanists and microbiologists (and their respective taxonomic codes) for almost two centuries: myxophyceae, cyanophyceae, blue**–**green algae, oxyphotobacteria, and cyanoprokaryotes are some of the epithets that have been used to name this evolutionarily distinct, easily recognizable group of photosynthetic microorganisms. But just when things seemed to have settled down in the last 30 years or so with the recognition of “cyanobacteria” as a bacterial clade at the rank of Phylum, we are confronted with a fundamentally different, deeper conundrum. Recent proposals based on phylogenetic studies of a set of marker genes common to most bacteria and archaea, have challenged not just *what* we ought to call them, but what cyanobacteria in fact *are*. Soo et al. (2014) argued that an entire group of lineages that do not share the main metabolic characteristics of cyanobacteria should be named cyanobacteria **–** or Cyanobacteriota as more recently modified (Soo et al. 2019). By including little‐known, but clearly nonphotosynthetic bacteria (Melainabacteria and Sericytochromatia) belonging to sibling clades into a new informal definition of the Phylum Cyanobacteria, the proposals do away with the correspondence between systematics and fundamental biological functionality. This newly conceptualized taxonomy has already made its way into widely used databases like Silva, while NCBI recognizes the close relationship between Melainabacteria and Cyanobacteria in a rankless group, but maintains the two as separate Phyla. And yet, since the time when the term cyanobacteria was coined by Stanier and colleagues (Stanier 1977), more than 23,000 published papers and numerous intellectual property documents have used it to describe prokaryotes capable of oxygenic photosynthesis. Of more than 500 cyanobacterial genomes that have been sequenced, all but a few are oxygenic. True, a few are able to carry out anoxygenic photosynthesis temporarily as the main photosynthetic mode when Photosystem II (PSII) is poisoned by hydrogen sulfide (Garcia‐Pichel and Castenholz 1990). And some, such as the symbiont *Candidatus Atelocyanobacterium thalassa* (also known as UCYN‐A) lost PSII, RuBisCO and photopigment genes(Tripp et al. 2010). But even these are clearly descendants of oxygenic phototrophs. This level of concordance between biological functionality, phylogeny, and nomenclature is a cherished, very useful rarity among bacteria. A direct benefit of this concordance is that identification of cyanobacterial sequences in a molecular survey immediately provides strong inference power about the nature of primary production in the ecosystem being studied. We contend here that these recent nomenclatural changes were unnecessary, ill‐timed, and are likely to cause much confusion, particularly when alternatives that preserve both the concordance between genomics and ecological function and information on the evolutionary relationships were at hand and would have constituted a more parsimonious approach. We propose a formal redefinition to redress the situation while leaving room to accommodate the newly discovered groups of heterotrophs.

The revolution in molecular phylogenetic approaches has had a profound effect on the description and classification of taxa. It facilitated identification of similarities and evolutionary relatedness, thereby enabling a pragmatic approach to inferring taxonomic hierarchies. In the past few years, new organisms, including uncultivated microorganisms, have been discovered and their genome sequences have been determined using cultivation‐independent techniques, including single‐cell genomics. The tree of life has dramatically changed, and is now replete with new branches, some of which are deep and suggest early diverging lineages of organisms (Hug et al. 2016). However, far from being a routine implementation of phylogenetic results, interpretation of these novel data in a wider biological context can often be subjective.

Soo et al. *(* 2014 *)* reported phylogenomic analyses of newly identified bacteria, Sericytochromatia and Melainabacteria, that are deeply branching sibling clades to cyanobacteria (Fig. 1). They concluded that Melainabacteria (Ley et al. 2005, Di Rienzi et al. 2013) and Sericytochromatia (Soo et al. 2014) share a common ancestor with cyanobacteria. The importance of this elegant genomic study and its robustly supported findings cannot be underestimated, because it provides the evolutionary context within which the cyanobacteria evolved as a distinct group. This major transition is marked by the development of oxygenic photosynthesis, a momentous feat in the evolutionary history of our planet that allowed cyanobacteria to tap into a virtually unrestricted source of electron donor for the reduction of CO_2_:water. Early on during the diversification of the phylum, cyanobacteria gave rise through symbioses to algal and plant forms (Keeling 2010). Phycology and Botany exist only because of this evolutionary cross‐talk. Even today, cyanobacteria remain one of the major drivers of primary productivity in the oceans and in vast expanses of arid lands, with a global biomass surpassing a thousand million metric tons (Garcia‐Pichel et al. 2003). It is thus exciting to delineate the biological context within which this evolutionary event first took place; one of likely anaerobic, fermentative bacteria. In our opinion regrettably, however, Soo et al. (2014) went on to rename the entire group Cyanobacteria or, once again more recently, Cyanobacteriota (Soo et al. 2019), changing the epithet for the traditional cyanobacteria to oxyphotobacteria, a term that has had little use and is considered a synonym to cyanobacteria (Castenholz 2015), demoting the Phylum to a Class, and obviating alternative options for a systematic treatment of these three phylogenetically distinct groups. The proposed nomenclature requires the use of awkward terminology such as “photosynthetic cyanobacteria” and “nonphotosynthetic cyanobacteria” (Blankenship 2017). The etimology of the root “cyano” in cyanobacteria in fact refers to the presence of photosynthetic antenna pigments, phycobiliproteins, that often give them a blue**–**green appearance. With the expanded meaning, one would have colorless blue**–**green bacteria. Here we wish to provide a biologically based, explicit alternative approach that would avoid unnecessary nomenclatural changes and the creation of a biologically heterogenous high order taxon that we consider to be of little practical use. An analogous example is the recent finding of *Rhodelphis;* gene and intron rich, phagotrophic flagellates that contain a genome‐lacking primary plastid and form a sister group to red algae (Rhodophyta) within the Archaeplastida (Gawryluk et al. 2019). This novel heterotrophic lineage would not in any sensible taxonomic scheme be considered as heterotrophic Rhodophyta because they lack the major attributes of red algae, namely, highly reduced nuclear gene inventories with few introns, lack of phagotrophy, absence of flagella and basal bodies, and a phycobilisome‐containing photosynthetic organelle (Bhattacharya et al. 2018). The term red alga has worldwide taxonomic import that is supported by their unique cell and genome biology.

**Figure 1:**
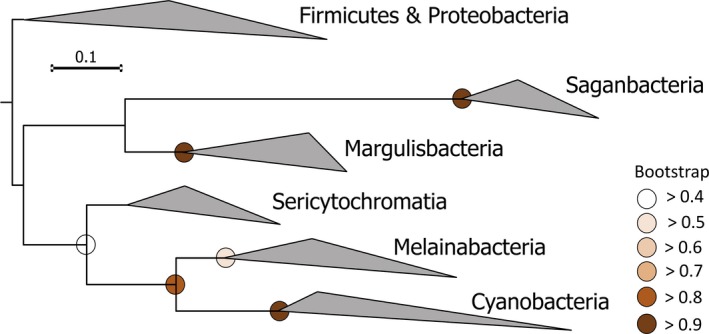
Maximum likelihood phylogenetic tree of the Cyanobacteria and sibling clades based on 16S rRNA sequences available in the Cydrasil curated database (https://github.com/FGPLab/cydrasil). Entries within each clade are collapsed triangles denoting maximal and minimal distances from their respective nodes. Nodes without color‐coding have bootstrap values less than 0.4.

Early studies reporting Melainabacteria (Ley et al. 2005, Di Rienzi et al. 2013) demonstrated that they lack all genes for thylakoid membrane‐bound electron transfer complexes such as PSI, PSII. and cytochrome *b*_*6*_ *f*, as well as those involved in aerobic respiration, and treated them as a sibling clade to cyanobacteria. Soo et al. (Soo et al. 2014) demonstrated that phylogenies obtained from sequences of a subset of shared genes placed Sericytochromatia, Melainabacteria, and Cyanobacteria into a single line of descent. Two other likely sibling clades have been subsequently described: Margulisbacteria and Saganbacteria (Matheus Carnevali et al. 2019). The relationships among these groups can be gleaned from a tree of the ribosomal 16S rRNA (Fig. 1), which reproduce those arrived at using phylogenomics. All of these groups are embedded within the larger (rankless) Terrabacteria (Battistuzzi and Hedges 2008) in the Domain Bacteria. None of these sibling clades other than the cyanobacteria, however, possess the genetic components to allow phototrophy or autotrophy. Although we do not contest the robustness of the phylogenetic and phylogenomic analyses, we wish to point out several factors that may result in a biased depiction of their relatedness with cyanobacteria. First, our knowledge of the new sibling groups to cyanobacteria is quite meager, and often indirectly obtained from inventorying known gene repertoires in their genomes. Only a handful of isolates have been studied, and many of the sequences and genomes used in these types of studies come from single‐cell genomes of environmental samples or metagenome‐assembled genomes, which provide only best guesses as to their metabolism or roles in the environment. It is conceivable that the current picture might soon change as more members or lineages are described, tipping the scales to a more intricate relationship or toward a more robust functional separation that is consistent with the currently known features. A network analysis approach shows that the evolution of cyanobacteria was complex, with metabolic diversity being inherited from both aerobes and anaerobes and that Melainabacteria might have shared a photosynthetic ancestor with cyanobacteria, a point not possible to prove because all photosynthesis genes are so far missing in *Melainabacter* (Harel et al. 2015), but perhaps present in some yet to be discovered member organisms. Second, and perhaps most relevant for our discussion, is the fact that molecular phylogenetic studies are based on the comparison of marker genes that are universally present among the organisms to be compared. In cases where a fundamental metabolic innovation takes place within the organismal set, the molecular sequences that encode the novel processes will, by definition, be excluded from the analyses. Hence, the comparisons will provide underestimates of the true evolutionary distance between the group that underwent the major innovation and its sister groups. This is clearly the case for cyanobacteria. All of its deeply rooted sister groups are completely missing genes for the photosynthetic apparatus as well as those for the Calvin**–**Benson cycle for carbon fixation. But photosynthetic proteins make up more than 50% of all proteins of cyanobacteria by weight, and typically 20**–**30% of all ORFs in cyanobacterial genomes are related to oxygenic photosynthesis. And yet the evolutionary investment in developing the machinery and photoautotrophic mode of life of cyanobacteria (see Overmann and Garcia‐Pichel 2013 for an account of what all this mode of life entails) bears no weight in the multigene phylogenies that use a subset of the genome information of common occurrence. Analyses of concatenated molecules also tend to average estimates of the evolutionary distance recorded in each element, which may not be coincident, resulting in estimates of evolutionary relationship that neglect estimate variance (Garcia‐Pichel et al. 2019). For example, the nitrogenase genes of Cyanobacteria are only distantly related to their homologs in Melainabacteria, yielding a polyphyletic relationship for the two phyla within the larger context of nitrogenase phylogeny. The trees are thus best interpreted as reconstructing a consensus phylogeny of the shared nonphotosynthetic “chassis” among these lineages. As an analogy, a child's carriage is more similar to an automobile than to a chariot when one looks at some of the major shared parts (2 axles and 4 wheels), even though they use fundamentally different power sources and are mechanistically worlds apart. Finally, although recent estimates of the timing of the split of cyanobacteria from nonphotosynthetic “protocyanobacteria” vary by more than one billion years (Shih et al. 2017, Garcia‐Pichel et al. 2019), the event can be considered under any model of evolution to be of ancient derivation. It is thus highly challenging to “prove” such deep relationships, because the ancestors, long gone, can never be analyzed. In sum, the phylogenetic relationships depicted in Figure 1 constitute an interesting piece of evidence that leads to an exciting hypothesis, one that likely underestimates the level of diversification among its phototrophic and nonphototrophic clades.

Traditional usage of the rank of phylum within the bacteria is not based on conventional standards but has rather evolved with the field as our knowledge of the extant microbial diversity accumulated, so as to accommodate clades that were sufficiently well‐delimited. The rank “phylum” finds no official place in the International Code of Nomenclature of Prokaryotes (Rule 5b; Parker et al. 2019), although it does in the International Code of Nomenclature for Algae, Fungi and Plants (Article 3; Turland et al. 2018). This mode of organic growth has resulted in a pragmatic, soft concept of the phylogenetic distance sufficient for naming new phyla, of which a large number are currently recognized (including some candidate phyla with few cultivated representatives). In looking at the plasticity of the concept on the basis of ribosomal RNA sequences, and using data from the exhaustive survey by Hug et al. (2016), the typical distance between an established phylum and its nearest neighbor hovers around 0.4**–**0.5 substitutions per site, but it varies between 0.1 (for example, between Gammaproteobacteria and Acidithiobacilli) and 0.6 (for example between *Deinococcus* /*Thermus* and Thermodesulfobacteria). The distance between Cyanobacteria and its closest sister clade, the Melainabacteria, in the same data set is about 0.3, not unlike that separating Actinobacteria and Gemmatimonatides, or that separating Betaproteobacteria from Gammaproteobacteria, or that between Synergistes and Thermotogae. Clearly the degree of relatedness of Melainabacteria and Cyanobacteria (the two most similar sibling clades among those discussed here) does not constitute a necessary reason to demote the traditional cyanobacteria to a class rank, or to lump them together with their sister clade into a single phylum. This is particularly the case, as discussed above, when these distances likely underestimate the true evolutionary difference between functionally divergent clades. Several research teams working on the phylogeny of cyanobacteria and its sibling clades saw no such need and treat each as a distinct group, keeping the phylum rank for cyanobacteria (Ley et al. 2005, Di Rienzi et al. 2013, Matheus Carnevali et al. 2019).

The issue at stake is thus not so much whether the groups evolved from a common phototrophic or nonphototrophic ancestor, or whether cyanobacteria acquired photosynthesis genes after diverging from the ancestral lineages, but whether such relationships justify using a term that has a widespread, established use in referring to a physiologically and ecologically coherent group. Regardless of the ancestral relationships, which are clearly deep and difficult to prove (Harel et al. 2015), it would be prudent to be conservative. We thus propose that the name Cyanobacteria be maintained for the lineage containing the evolutionarily related organisms that acquired the oxygenic phototrophic trait, and a new epithet be coined for the larger group of clades to avoid further confusion if things are left as they are now. In view of the expanding dynamics of discovery, a question of relevance is if one should include Margulisbacteria and Saganbacteria in that definition. More work will be needed to find the answer. Temperance is thus on the side of eventual taxonomic stability, and we refrain here from doing so. We have to be mindful that nomenclature should pragmatically serve not only those familiar with the intricacies and subtleties of systematics, but also the more casual practitioners of science in all other relevant basic and applied disciplines, for which simplicity and congruency is a definite asset. In this regard, the willingness of sequence databases to timely correct the situation is paramount. In order to enable the correction, we provide a formal definition of the Phylum under the International Code for Nomenclature of Algae, Fungi, and Plants:

**Phylum Cyanobacteria** *: (latinized from Greek Kyanos [Κ*ψανοσ*], blue* **–** *green, after the coloration imparted to many of its species by phycocyanin, and from latinized Greek Bacterion [Β*αχτεριον*], staff or cane, denoting an elongated shape). A phylogenetic lineage of organisms in the domain bacteria able to carry out oxygenic photosynthesis with water as an electron donor and to reduce carbon dioxide as a source of carbon, or those secondarily evolved from such organisms*.

*Synonyms: blue* **–** *green algae, cyanophyta, oxyphotobacteria, oxychlorobacteria, myxophyceae*
